# Salvianolic acid B ameliorates myocardial fibrosis in diabetic cardiomyopathy by deubiquitinating Smad7

**DOI:** 10.1186/s13020-023-00868-9

**Published:** 2023-12-10

**Authors:** Hong Luo, Lingyun Fu, Xueting Wang, Yini Xu, Ling Tao, Xiangchun Shen

**Affiliations:** 1 The State Key Laboratory of Functions and Applications of Medicinal Plants, School of Basic Medical Sciences, Guizhou Medical University, Ankang Road, Guian New District, Guizhou, 561113 China; 2 The Key Laboratory of Optimal Utilization of Natural Medicine Resources, School of Pharmaceutical Sciences, Guizhou Medical University, Ankang Road, Guin New District, Guizhou, 561113 China; 3 The Experimental Animal Center of Guizhou Medical University, Guizhou Medical University, Ankang Road, Guian New District, Guizhou, 561113 China

**Keywords:** Salvianolic acid B, Diabetic cardiomyopathy, Myocardial fibrosis, TGF-β1 signaling pathway, Deubiquitinating Smad7

## Abstract

**Background:**

Salvianolic acid B (Sal B), a water-soluble phenolic compound derived from *Salvia miltiorrhiza* Bunge, is commonly used in Traditional Chinese Medicine to treat cardiovascular disease. In our previous study, Sal B protected against myocardial fibrosis induced by diabetic cardiomyopathy (DCM). This study aimed to investigate the ameliorative effects and potential mechanisms of Sal B in mitigating myocardial fibrosis induced by DCM.

**Methods:**

Various methods were used to investigate the effects of Sal B on myocardial fibrosis induced by DCM in vivo and in vitro. These methods included blood glucose measurement, echocardiography, HE staining, Masson’s trichrome staining, Sirius red staining, cell proliferation assessment, determination of hydroxyproline levels, immunohistochemical staining, evaluation of fibrosis-related protein expression (Collagen-I, Collagen-III, TGF-β1, p-Smad3, Smad3, Smad7, and α-smooth muscle actin), analysis of *Smad7* gene expression, and analysis of Smad7 ubiquitin modification.

**Results:**

The animal test results indicated that Sal B significantly improved cardiac function, inhibited collagen deposition and phenotypic transformation, and ameliorated myocardial fibrosis in DCM by upregulating Smad7, thereby inhibiting the TGF-β1 signaling pathway. In addition, cell experiments demonstrated that Sal B significantly inhibited the proliferation, migration, phenotypic transformation, and collagen secretion of cardiac fibroblasts (CFs) induced by high glucose (HG). Sal B significantly decreased the ubiquitination of Smad7 and stabilized the protein expression of Smad7, thereby increasing the protein expression of Smad7 in CFs and inhibiting the TGF-β1 signaling pathway, which may be the potential mechanism by which Sal B mitigates myocardial fibrosis induced by DCM.

**Conclusion:**

This study revealed that Sal B can improve myocardial fibrosis in DCM by deubiquitinating Smad7, stabilizing the protein expression of Smad7, and blocking the TGF-β1 signaling pathway.

## Background

Since the 1950s, the incidence of diabetes mellitus (DM) has increased dramatically worldwide and has become a major public health concern. In China, there were 140.9 million diabetes patients in 2021, which means one in five people to be diabetic [1]. Diabetic cardiomyopathy (DCM), caused by DM, is one of the most common causes of morbidity and mortality globally, contributing to over 50% of diabetes-related deaths [2]. Myocardial fibrosis is the primary pathological change in DCM [3, 4]. The pathological features of myocardial fibrosis involve excess deposition of extracellular matrix (ECM) [5], resulting in the thickening and stiffness of the cardiac walls increased, leads to heart failure, cardiac dysfunction, arrhythmias, and heart attacks, and eventually cardiovascular death. [6, 7]. Cardiac fibroblasts (CFs) are one of the main effectors in fibrotic scar formation in the heart, owing to their role in ECM turnover [8]. When myocardial fibrosis occurs, CFs proliferation, migration, phenotypic transformation, and collagen secretion significantly increase [9, 10]. The differentiation of CFs into myofibroblasts is a crucial process in myocardial fibrosis, with α-smooth muscle actin (α-SMA) considered a marker of myofibroblast differentiation [11]. Myofibroblasts play key roles in collagen deposition during fibrogenesis [12]. Inhibiting the proliferation, migration, phenotypic transformation, and collagen secretion of CFs is a dominant strategy for attenuating myocardial fibrosis [13, 14]. Additionally, the TGF-β1 signaling pathway is the most important target in DCM fibrosis pathogenesis [15]. Inhibition of the TGF-β1 signaling pathway can significantly ameliorate myocardial fibrosis in DCM [16]. Mothers Against decapentaplegic homolog 7 (Smad7) is a crucial negative feedback factor in the TGF-β1 signaling pathway [17, 18]. Smad7 can block the TGF-β1 signaling pathway by inhibiting the phosphorylation of receptor-regulated Smads, such as Smad3 [19]. Substantial evidence has shown that downregulating the expression of Smad7, myocardial fibrosis significantly deteriorate [20]. However, there are no effective treatment options for myocardial fibrosis induced by DCM currently. Salvinolic acid B (Sal B) is a phenolic compound found in *Salvia miltiorrhiza* Bunge [21], which is commonly used to treat cardiovascular diseases in Traditional Chinese Medicine. Sal B consists of three tanshinol molecules and one caffeic acid molecule [22]. It exhibits antioxidant properties because its structure contains multiple phenolic hydroxyls [23]. Our previous studies have shown that Sal B exhibits potential protective effects against myocardial fibrosis induced by DCM. In this study, we investigated the ameliorative effects and potential mechanism of Sal B on myocardial fibrosis in DCM in vivo and in vitro, providing evidence that Sal B ameliorates myocardial fibrosis induced by DCM, thus encouraging the use of Traditional Chinese Medicine in clinical applications.

## Materials and methods

### Chemicals and reagents

Sal B (purity 98%) was purchased from Chengdu Puruifa Technology Co., Ltd. (Chengdu, China). A high-fat and high-sucrose diet (60% k cal fat, D12492i) was obtained from Research Diets Co., Ltd. (New Jersey, USA). The Streptozotocin was purchased from Solaibao Biological Technology Co., Ltd. (Beijing, China). Metformin was purchased from Shanghai Yisheng Biotechnology Co., Ltd. (Shanghai, China). A mouse insulin ELISA test kit was purchased from Shanghai Overtone Biotechnology Co., Ltd. (Shanghai, China). Cycloheximide (CHX) was obtained from Master of Bioactive Molecules Biotechnology Co. Ltd. (New Jersey, USA). Vimentin, GAPDH, TGF-β1, Col-I, Col-III, and α-SMA antibodies were obtained from Wuhan Boster Biological Technology Co., Ltd. (Wuhan, China). The ubiquitin antibody and coralite 594-conjugated goat anti-rabbit IgG was obtained from Proteintech Co., Ltd. (Wuhan, China).

### Animal protocol

Healthy male C57BL/6J mice (body weight, 18–20 g) were obtained from Sibeifu Biotechnology Co., Ltd. (Beijing, China) and housed under controlled temperature (25 °C) and photoperiod (12 h:12 h light-dark cycle) conditions. The animal protocols were conducted in accordance with the NIH Guide for the Care and Use of Laboratory Animals (NIH Publication 85-23, revised 1996). The mice were randomly divided into control (n = 20) and DCM (n = 100) groups. During the 3-month period, DCM mice were fed a high-fat and high-sucrose diet, while control mice were fed a normal chow diet. Glucose tolerance tests (GTTs) and enzyme-linked immunosorbent assay (ELISA) were performed to measure the blood glucose level and serum fasting insulin level to confirm that the mice had developed insulin resistance. A mouse model of type 2 diabetes was established using streptozotocin (30 mg/kg/time, intraperitoneal injection every other day). Mouse blood glucose levels were measured after the last induction for 1 month when blood glucose levels were stably maintained. Type 2 diabetes was confirmed by fasting glucose levels above 11.1 mmol/L [24]. The type 2 diabetic mice were randomly divided into four groups: diabetic cardiomyopathy model group (DCM, normal saline 10 mL/kg/day, ig), Sal B low-dose group (Sal B. L, 1.5 mg/kg/day, ig), Sal B high-dose group (Sal B. H, 3 mg/kg/day, ig), metformin group (Met, 200 mg/kg/day, ig), mice fed a normal chow diet were used as the control group (Control, normal saline 10 mL/kg/day, ig). All interventions were administered by gavage 6 days/week. The ejection fraction (EF) of the control and DCM groups was assessed monthly, and the DCM model was successfully established when the EF of the DCM group was significantly lower than that of the control group [25].

### Echocardiographic recordings

Echocardiographic measurement was performed at the end of the study period. A 2D M-mode echocardiograph equipped with a 30 MHz linear transducer echocardiographic system (Vinno, China) was used to evaluate cardiac morphology and function [26]. The key measurements were evaluated: left ventricular end diastolic volume (LVEDV), left ventricular end systolic volume (LVESV), fractional shortening (FS), and ejection fraction (EF).

### Histological analysis

At the end of the experiment, the mice were anesthetized by inhaling isoflurane, the hearts of the mice in each group were collected, part of the heart tissue was used for analysis of protein expression, and part of the tissue was fixed in 4% paraformaldehyde solution in a 0.1 M phosphate buffer for 48 h. Following fixation, tissues were dehydrated and embedded in paraffin. Tissue sections of 4 μm thickness were prepared and mounted on glass slides. To evaluate the extent of fibrosis in the heart tissues, histological staining techniques such as hematoxylin and eosin (HE) staining, Masson’s trichrome staining, and Sirius red staining as described previously were used [27]. Quantification of fibrotic regions and total tissue area was performed using Image-Pro Plus (version 6.0). The degree of fibrosis was determined by calculating the ratio between the area exhibiting fibrosis and the total tissue area, expressed as a percentage [% fibrosis = (area exhibiting fibrosis/total area) * 100].

### Isolation and culture of neonatal rat CFs

Neonatal rat CFs were obtained using the tryptic digestion method. Briefly, hearts from neonatal Sprague Dawley rats aged 1–3 d old were digested with 0.125% trypsin (Solaibao, T1300) at 4 °C for 6 h, followed by a 5 min digestion at 37 °C. This digestion process was repeated 4–6 times. To separate CFs from cardiomyocytes, a differential adhesion technique was employed for 90 min, as CFs and cardiomyocytes adhere to surfaces at different rates [28]. The isolated CFs were cultured in Dulbecco’s modified Eagle’s medium supplemented with 15% fetal bovine serum at 37 °C in a 5% CO_2_ atmosphere. The resulting primary neonatal rat CFs were used for subculturing. The second or third passage of CFs was used for further experiments.

### Immunofluorescence staining

Immunofluorescence staining of CFs with anti-vimentin and anti-Smad7 antibodies was performed to identify cells and observe the effect of Sal B on Smad7 expression as described previously [29]. CFs were cultured in 6-well plates for 24 h and incubated with different concentrations of glucose and Sal B medium for 24 h or not. Cells were fixed with 4% paraformaldehyde for 30 min. After washing three times using PBS, the plate was infiltrated with 0.2% Triton-X100 (Solaibao, IT9100) for 20 min and blocked with 2% BSA (Solaibao, A8010) for 30 min. Then, the cells were incubated with primary antibodies (vimentin and Smad7, 1:500) overnight at 4 °C, washed three times using PBS and incubated with a secondary antibody (coralite 594-conjugated goat anti-rabbit IgG, 1:500) for 1 h in the dark. Nuclei were stained with DAPI (Solaibao, C0065) for 5 min. Images of cells were captured by fluorescence microscopy (Leica, Germany). Quantification of fluorescence intensity was performed using Image-Pro Plus software (version 6.0).

### Cell proliferation evaluation

This study utilized the xCELLigence Real-Time Cell Analyzer system (Agilent, USA), which employs sensor impedance technology to assess the cell status through a unitless parameter known as the cell index. The cell index reflects cell status by measuring the relative changes in electrical impedance in the presence and absence of cells in the wells [30]. The xCELLigence Real-Time Cell Analyzer was used to optimize the concentration of high glucose (HG)-induced CF proliferation and evaluate the inhibitory effect of Sal B on the proliferation of CFs induced by HG. Briefly, CFs were seeded onto E-plates (Agilent, USA) for 24 h at 37 °C in 100 µL of medium. The cell index of CFs was measured every 15 min after incubation with different concentrations of glucose and Sal B medium for 120 h. To eliminate any influence of differences in osmotic pressure changed by HG, mannitol (Solaibao, IM0040) was used as a control group of osmotic pressure. The cell morphology was observed by HE staining as described previously. Briefly, CFs were seeded onto 12-well plates for 24 h, added to different concentrations of glucose and Sal B medium for 24 h, and washed with PBS three times. CFs were fixed with 4% paraformaldehyde for 30 min after washing three times using PBS. HE staining was performed using an HE Staining Kit (Solaibao, G1120) according to the manufacturer’s protocols. Images of cells were captured by inverted microscope (Olympus, Japan).

### Cell scratch assay

CF migration was assessed by a cell scratch assay. CFs were cultured in 6-well plates for 24 h, the scratch area was made using a 100 µL pipette tip, 40 mM glucose and different concentrations of Sal B medium were added, and the plates were incubated for 24 h. Cell scratches were imaged via microscopy at 0 and 24 h. From five averaged regions, the width of the cell scratch area was measured by Image-Pro Plus (version 6.0). Migration distances after 24 h were subtracted from baseline distances. The relative migrating distance of the cells was measured as the distance of cell migration and the wound distance at 0 h, expressed as a percentage [% migration = [(wound distance at T0 h − wound distance at T24 h)/wound distance at 0 h)] × 100%.

### Myocardial hydroxyproline concentration

The hydroxyproline (Hyp) content of the cell supernatants was quantified using a Hyp assay kit (Jiancheng, A030) according to the manufacturer’s protocols as described previously [31]. Microplate reader ELX800 (Biotek, USA) was used to measure the OD values of the samples at 550 nm. The results were expressed as µg/mL of total protein.

### Western blotting analysis

Protein samples were extracted from mouse heart tissues and CFs. Briefly, myocardial tissue was lysed in 200 µL of ice-cold RIPA buffer (Solaibao, R0010) containing 0.2 mM PMSF (Solarbio, P0100). CFs were lysed using the same lysate. BCA Protein Assay Kits (Solarbio, PC0020) were used to determine the total protein concentration. Proteins were separated by 12% sodium dodecylsulfate-polyacrylamide gel electrophoresis (Epizyme Biotech, PG213) and then transferred from the gel to polyvinylidene difluoride membranes (Millipore, IPVH00010). An overnight incubation with primary antibodies (Col-I, Col-III, α-SMA, TGF-β1, Smad3, Smad7, 1:1000) was performed after blocking the membrane with 5% bovine serum albumin. The membranes were incubated with the appropriate secondary antibody (1:10,000) for 1 h at room temperature (20 ± 5 °C). An ECL kit (Millipore, WBKLS0500) was used to detect bands. GAPDH (1:10,000) was used as a reference for total cell protein, and protein bands were scanned and analyzed using Image-Pro Plus (version 6.0) for gray values. Relative protein expression = gray value of the target proteins/gray value of the GAPDH protein bands.

### RNA extraction and quantitative real-time polymerase chain reaction

qRT-PCR was used to investigate the mechanisms by which Sal B enhances the expression of Smad7 [32, 33]. In accordance with the standard protocol, total cellular RNA was extracted from CFs using the Total RNA Kit I. RNA (Omega, R6834) was reverse-transcribed into complementary DNA (cDNA) using the PrimeScript™ RT reagent kit (Takara, RR037A) and SimpliAmp Thermal Cycler (Life Technologies,USA). CFX Manager 3.0 A Real-Time PCR System (Bio-Rad, USA) was used to perform quantitative real-time PCR using SYBR® Premix Ex TaqTM II (Takara, RR820A). GAPDH was used as an internal control for mRNA expression. The primers were designed as follows (Table 1):

**Table 1:** Primer sequences for qRT-PCR

Gene	Forward 5′–3′	Reverse 5′–3′
Smad7	GTGGCATACTGGGAGGAGAA	AGCTGACTCTTGTTGTCCGA
GAPDH	GACATGCCGCCTGGAGAAAC	AGCCCAGGATGCCCTTTAGT

### Immunoprecipitation analysis of Smad7

The inhibitory effect of Sal B on the ubiquitination of Smad7 was investigated by immunoprecipitation using the Classic Magnetic Protein A/G IP/Co-IP Kit (Epizyme Biotech, YJ201) [34, 35]. CFs were divided into four groups: control group (5.5 mM glucose), HG group (40 mM glucose), HG (40 mM glucose) + Sal B (25 µM) groups, and IgG negative control group, incubated for 24 h. The proteasome inhibitor MG132 (Beyotime, S1748) was used at a final concentration of 2 µM. Cells were lysed in 500 µL of lysis buffer. Total protein was extracted from the cultured cells using a previously described method. The extracted proteins were incubated with Smad7 antibodies, and the negative control group was incubated with rabbit IgG (Beyotime, A7016). The mixture was incubated at 4 °C overnight with gent shaking to form antibody-antigen complexes. To obtain the antigen-antibody-magnetic bead mixture, pretreated magnetic beads were added to the antigen-antibody mixture and incubated overnight at 4 °C. Finally, the antigen-antibody complex was eluted from the mixture, and the beads were boiled for 5 min. The supernatant was collected for western blot analysis. Smad7 was detected using an anti-ubiquitin antibody (1:1000).

### Statistical analysis

The data were analyzed using GraphPad Prism statistical software (version 5.0). Data are presented as the mean ± standard deviation (SD). To assess the significant differences among the groups, an analysis of variance (ANOVA) with Tukey’s post hoc test was conducted. Statistical significance was set at *p* < 0.05.

## Results

### Sal B improved cardiac function in DCM mice

**Fig. 1:**
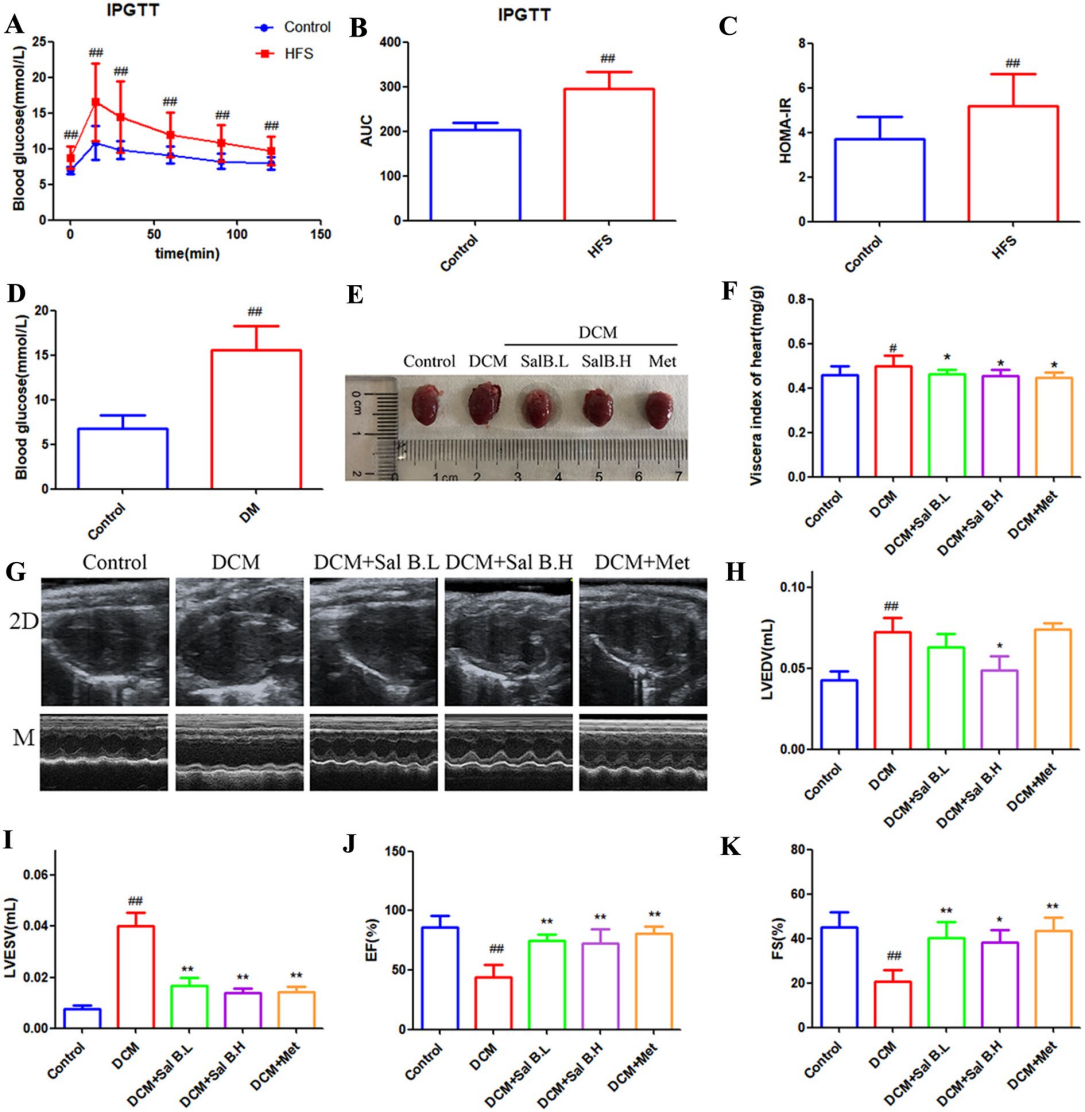
Effects of Sal B on cardiac dysfunction in DCM mice. **A** Intraperitoneal glucose tolerance test (IPGTT); **B** area under curve (AUC) of the blood glucose; **C** insulin resistance was observed in rats after HFS diet feeding for 3 months (HOMA-IR); **D** blood glucose level of type 2 diabetes model; **E** anatomical image of DCM mice; **F** Heart viscera index of DCM mice; **G** representative images of 2D and M-mode echocardiography; **H**–**K** quantitative results of LVESV, LVEDV, EF, FS of mice heart. N = 8. Data are presented as the mean ± SD. One-way ANOVA was employed for comparisons among multiple groups, followed by Tukey’s test. ^#^*p* < 0.05, ^##^*p* < 0.01 vs. the control group, *p < 0.05, **p < 0.01 vs. the DCM group

The type 2 diabetes model was established using a high-fat and high-sucrose diet combined with low-dose STZ. Compared to the control group, mice fed a high-fat and high-sucrose diet showed significantly impaired glucose tolerance and insulin resistance (Fig. 1A–C). After STZ induction, the blood levels of glucose increased remarkably (Fig. 1D), and type 2 diabetes was confirmed by the presence of blood glucose concentrations > 11.1 mmol/L. The type 2 diabetes model was considered successfully established. Compared to the control group, EF decreased after 8 weeks of placement, and the DCM mouse model was reproduced successfully (Fig. 1J). Left ventricular hypertrophy and dilated left ventricles were observed in DCM mouse hearts (Fig. 1E, G). The heart viscera index of DCM mice was significantly increased in the DCM group (Fig. 1F). However, after Sal B and Met treatment, heart hypertrophy and left ventricular dilation were significantly improved (Fig. 1E, G), and the heart viscera index of Sal B- and Met-treated mice was significantly lower than that of the DCM group (Fig. 1F). Cardiac ultrasound results showed that in DCM mouse hearts, the LVESV and LVEDV were significantly increased, and the EF and FS were significantly decreased, which improved after Sal B and Met treatment (Fig. 1H–K). Taken together, these results indicated that Sal B significantly improved the cardiac function of DCM mice.

### Sal B inhibits inflammatory cell infiltration, collagen deposition, phenotypic transformation, and the TGF-β1 signaling pathway in DCM mice

**Fig. 2:**
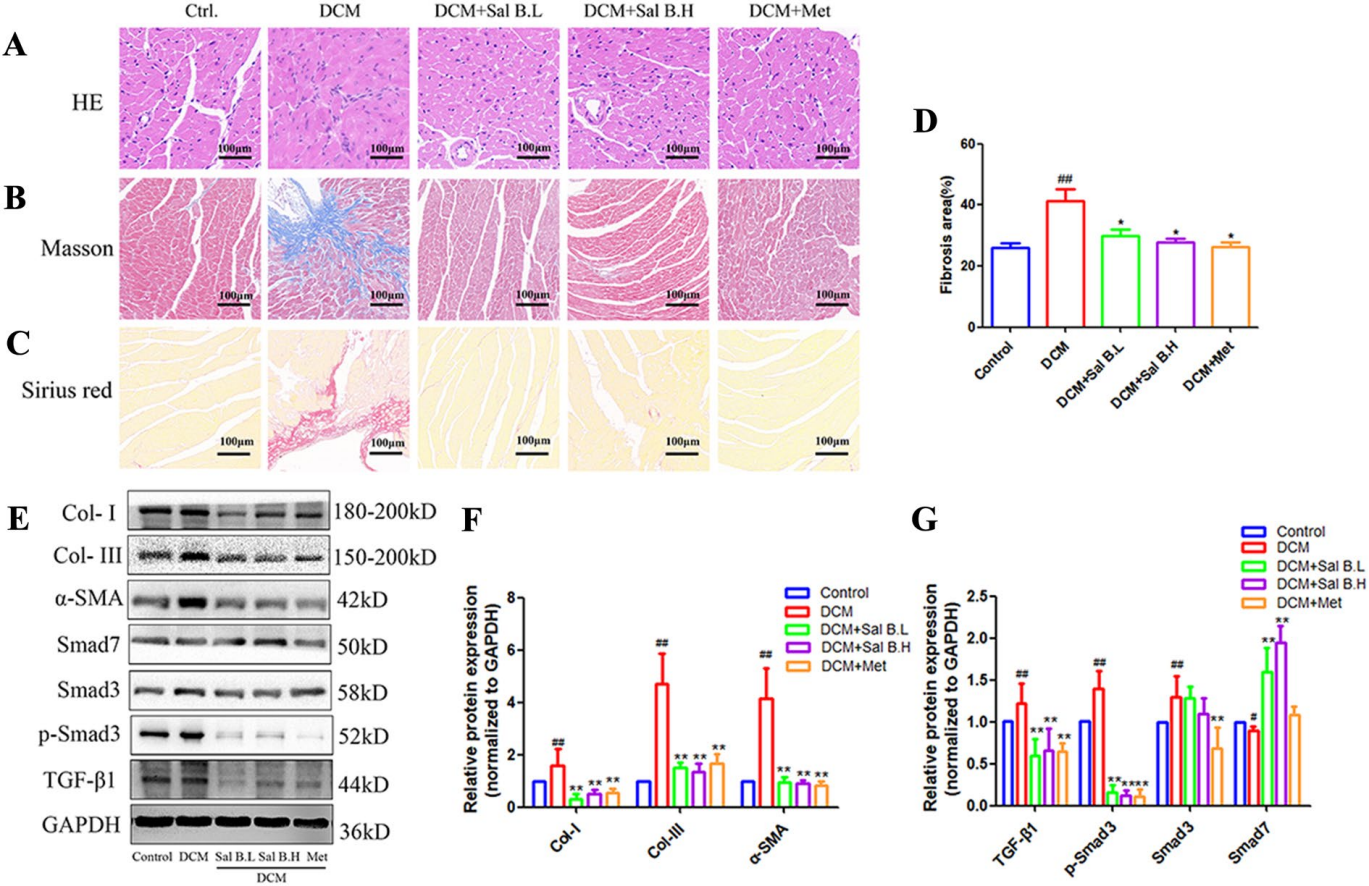
Effects of Sal B on inflammatory cell infiltration and collagen deposition in DCM mice. **A**–**C** Hematoxylin and eosin (HE), Masson staining and Sirius red staining of heart tissue; **D** quantitative Masson trichromatic analysis of collagen area; **E** expression level of myocardial fibrosis-related proteins; **F**, **G** quantitative results of protein expression. The expression of all proteins was normalized to GAPDH. N = 8. Data are presented as the mean ± SD. One-way ANOVA was employed for comparisons among multiple groups, followed by Tukey’s test. ^#^*p* < 0.05, ^##^*p* < 0.01 vs. the control group, *p < 0.05, **p < 0.01 vs. the DCM group

Histological examinations revealed significant myocardial hypertrophy and inflammatory cell infiltration in DCM mice; however, after treatment with Sal B and Met, a notable improvement in myocardial hypertrophy and inflammatory cell infiltration was observed (Fig. 2A). Myocardial fibrosis is characterized by excessive deposition of fibrotic extracellular matrix proteins, especially collagen I (Col-I) and collagen-III (Col-III) [36]. Masson and Sirius red staining showed a large amount of collagen deposition in the myocardial tissue of mice with DCM. Sal B and Met treatment significantly decreased collagen deposition (Fig. 2B, C). Cardiac fibrosis was quantified by calculating the ratio between the area exhibiting fibrosis and the total tissue area after Masson trichrome staining. The results showed that the ratio increased significantly in the myocardial tissue of the DCM group compared with the Sal B and Met groups (Fig. 2D). Western blot results consistently showed that the expression of Col-I and Col-III was significantly upregulated in the myocardial tissue of DCM mice. After Sal B and Met treatment, the expression of Col-I and Col-III in mouse myocardial tissue was significantly downregulated (Fig. 2E, F), indicating that Sal B can significantly decrease collagen secretion. Myofibroblasts produce more collagen during fibrogenesis and are responsible for collagen deposition [37]. The differentiation of fibroblasts into myofibroblasts was assessed by Western blot analysis for α-SMA expression. Western blotting results showed that the expression of α-SMA in the myocardial tissue of DCM mice was significantly upregulated. After Sal B and Met treatment, α-SMA expression in mouse myocardial tissue was significantly downregulated (Fig. 2E, F), which indicated that Sal B significantly decreased phenotypic transformation in DCM mice. In addition, western blotting results showed that the expression of TGF-β1, p-Smad3 and Smad3 in the myocardial tissue of DCM mice was significantly upregulated and that of Smad7 was significantly downregulated (Fig. 2E, G). After Sal B and Met treatment, the expression of TGF-β1, p-Smad3, and Smad3 in mouse myocardial tissue was significantly downregulated, whereas Smad7 was significantly upregulated (Fig. 2E, G), which indicated that Sal B significantly improved myocardial fibrosis in DCM mice by upregulating Smad7 to inhibit the TGF-β1 signaling pathway.

### Identification and morphology of primary neonatal rat CFs

**Fig. 3:**
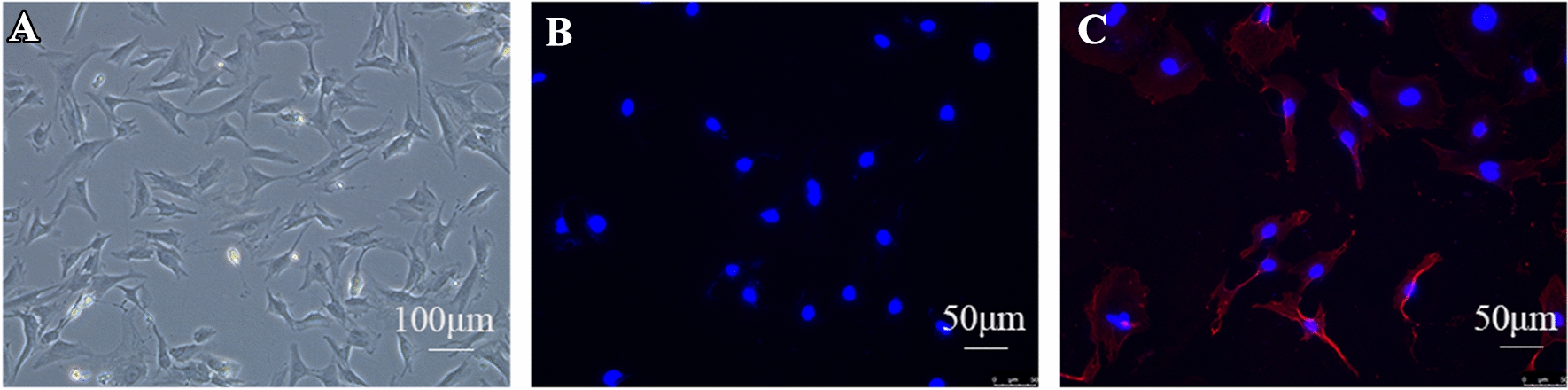
Identification and morphology of primary neonatal rat CFs. **A** CFs grow after three days; **B** negative control; **C** vimentin-positive cells

Under the microscope, neonatal rat CFs appeared as spindle-shaped cells with multiple projecting processes (Fig. 3A). Vimentin, a specific marker for CFs, was detected using immunocytochemistry, revealing its filamentous structure [38]. Control cells treated with PBS instead of the anti-vimentin antibody showed no red fluorescence and only blue nucleus staining (Fig. 3B). In contrast, primary cells stained with the anti-vimentin antibody exhibited strong red fluorescence along with blue nucleus staining, confirming the presence of vimentin (Fig. 3C). Quantitative analysis demonstrated that 95% of the cells were positive for vimentin.

### Sal B inhibits the proliferation of CFs induced by HG

**Fig. 4:**
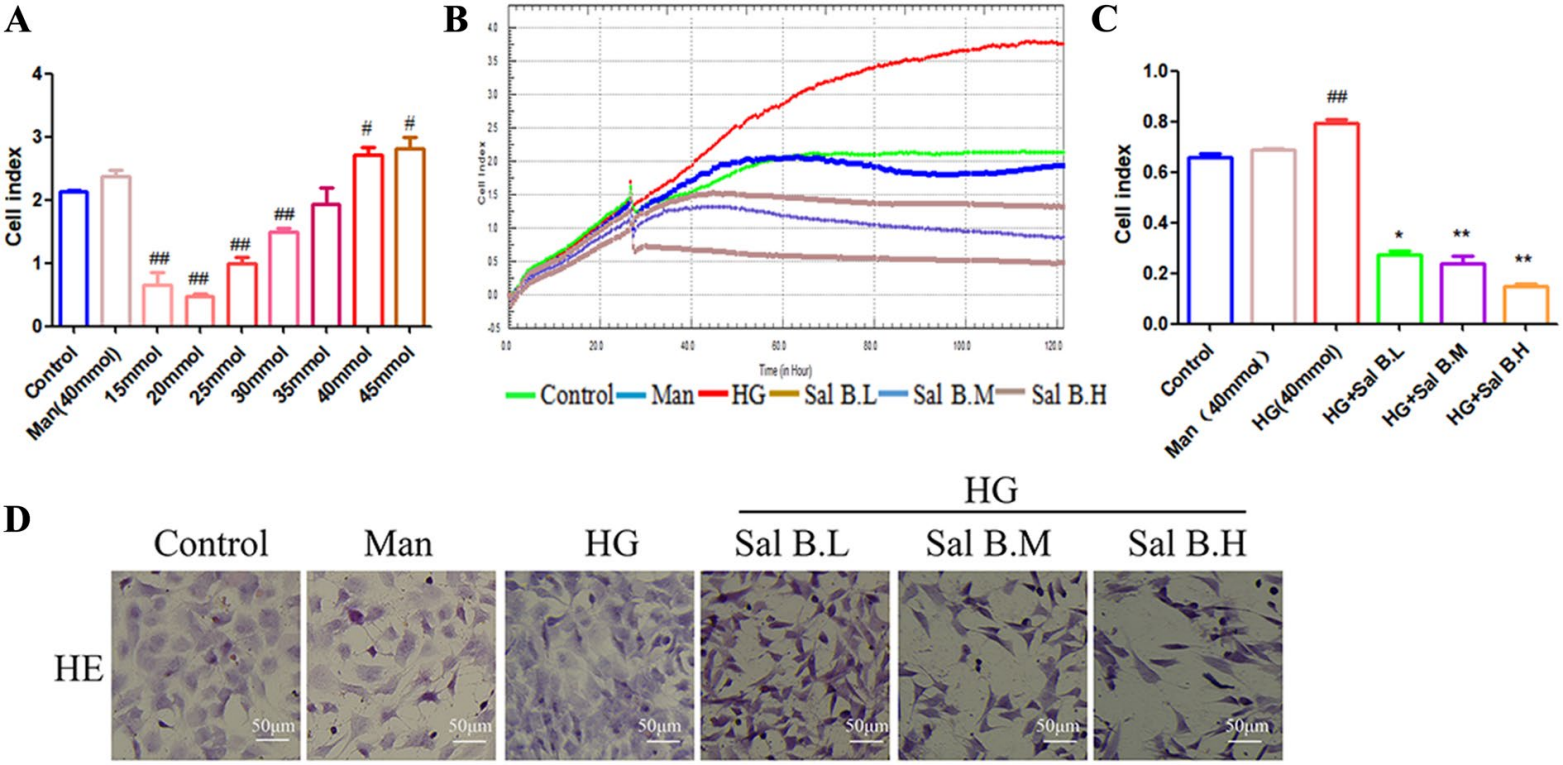
Effects of Sal B on the proliferation of CFs induced by HG. **A** Dose-effect relationship of cardiac fibroblast proliferation induced by HG; **B** effect of Sal B on the HG-induced CF cell index by an xCELLigence Real-Time Cell Analyzer (0–120 h); **C** effect of Sal B on the HG-induced CF cell index (24 h); **D** HE staining. The cell experiment was repeated three times. Data are expressed as the mean ± SD. One-way ANOVA was applied for comparisons among multiple groups, followed by Tukey’s test. ^#^*p* < 0.05, ^##^*p* < 0.01 vs. the control group, *p < 0.05, **p < 0.01 vs. the DCM group

Compared to the control group, the proliferation of CFs was significantly increased by 40 mM and 45 mM glucose. Previous studies suggested that 40 mM glucose is the optimal concentration for inducing CF proliferation (Fig. 4A) [39]. The results of cell proliferation and HE staining indicated that Sal B (12.5–50 µM) significantly inhibited the proliferation of CFs induced by HG (Fig. 4B–D).

### Sal B decreased the migration and hydroxyproline secretion of CFs

**Fig. 5:**
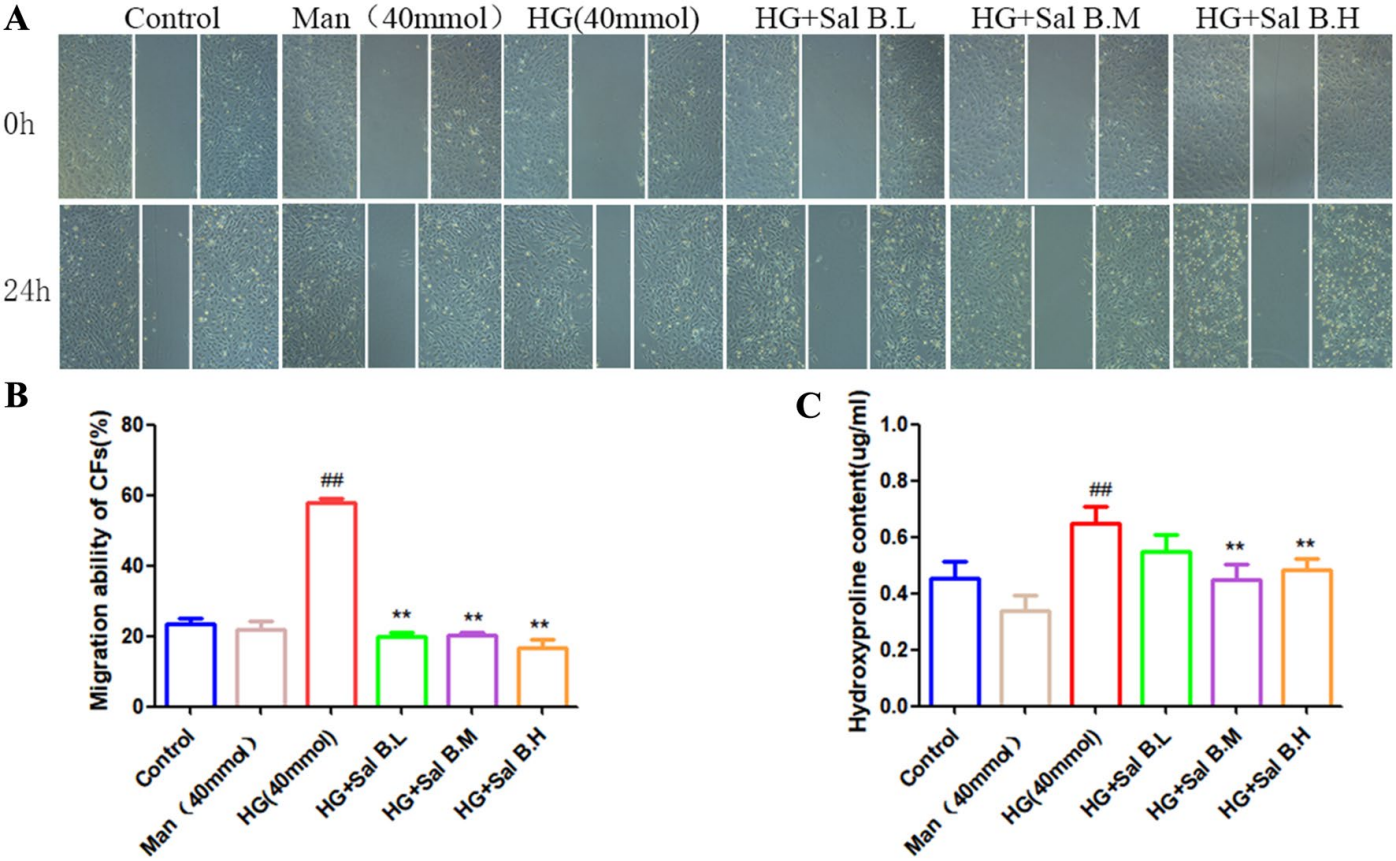
Effects of Sal B on migration and hydroxyproline secretion of CFs induced by HG. **A** Effects of Sal B on the migration of neonatal rat CFs induced by HG (24 h); **B** quantitative migration results; **C** effect of Sal B on hydroxyproline secretion from CFs induced by HG. The cell experiment was repeated three times. Data are expressed as the mean ± SD. One-way ANOVA was applied for comparisons among multiple groups, followed by Tukey’s test. ^#^*p* < 0.05, ^##^*p* < 0.01 vs. the control group, *p < 0.05, **p < 0.01 vs. the DCM group

Cell scratch assays were performed to determine the effects of Sal B on migration CFs induced by HG. Compared to the control group, HG significantly increased the migration of CFs, and Sal B significantly inhibited the migration ability of CFs induced by HG (Fig. 5A, B). Myocardial collagen content was estimated by measuring myocardial hydroxyproline concentrations. Compared to the control group, HG significantly increased the secretion of hydroxyproline by CFs induced by HG. Sal B significantly inhibited the secretion of hydroxyproline from CFs induced by HG (Fig. 5C).

### Sal B inhibited myocardial fibrosis and TGF-β1 signaling pathway-related proteins and increased the expression of Smad7 in CFs induced by HG

**Fig. 6:**
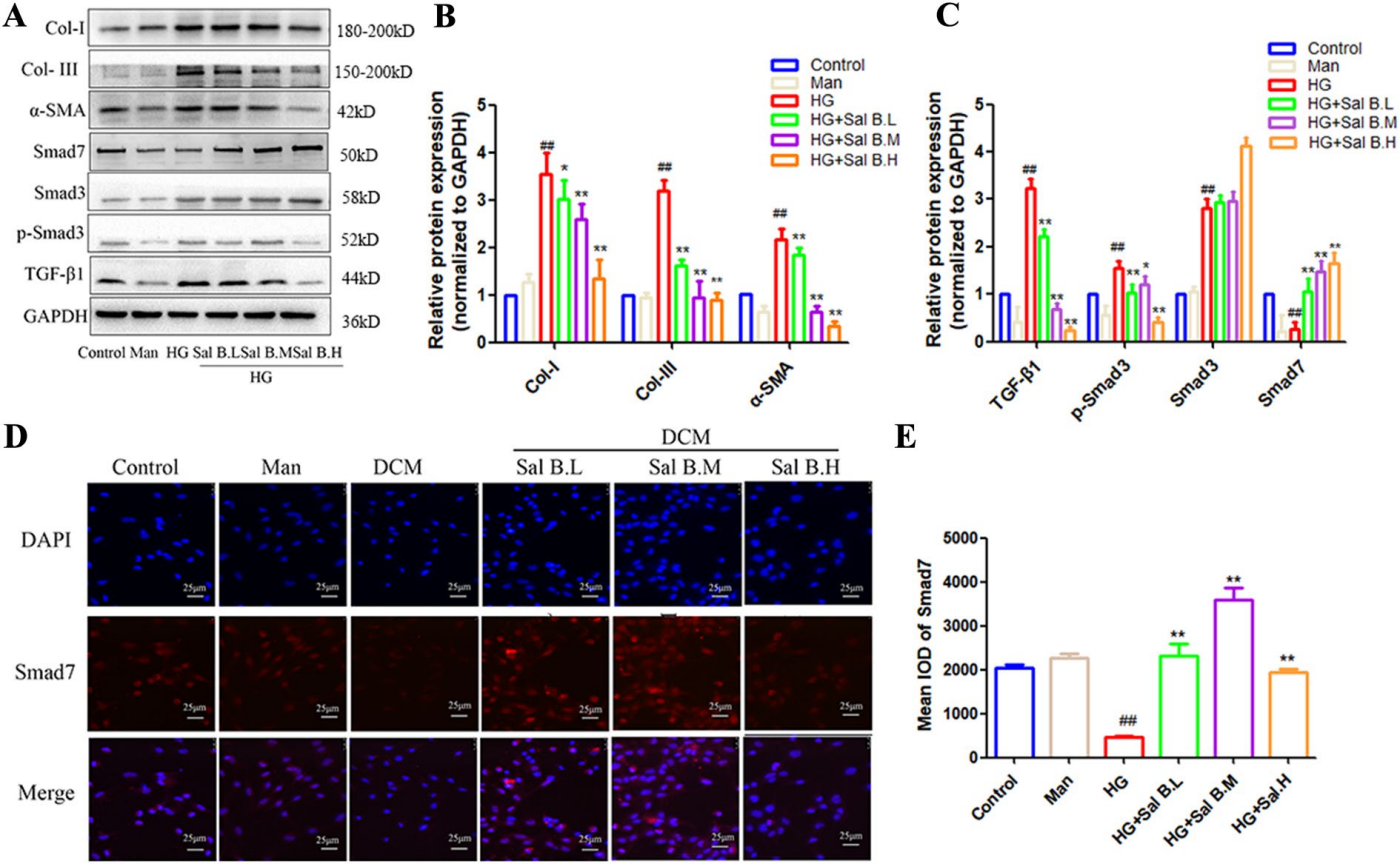
Effects of Sal B on the expression of myocardial fibrosis-related proteins in CFs induced by HG. **A** Expression level of myocardial fibrosis and TGF-β1 signaling pathway related-proteins; **B** quantitative analysis of myocardial fibrosis proteins related-expression. The expression of all proteins was normalized to GAPDH. **C** Quantitative analysis of TGF-β1 signaling pathway-related proteins. The expression of all proteins was normalized to GAPDH. **D** Effects of Sal B on Smad7 expression in CFs induced by HG. **E** Quantitative analysis of Smad7 expression. The cell experiment was repeated three times. Data are expressed as the mean ± SD. One-way ANOVA was applied for comparisons among multiple groups, followed by Tukey’s test. ^#^*p* < 0.05, ^##^*p* < 0.01 vs. the control group, *p < 0.05, **p < 0.01 vs. the DCM group

Compared to the control group, the protein levels of Col-I, Col-III, TGF-β1, p-Smad3, Smad3 and α-SMA in CFs induced by HG were significantly increased, and Sal B significantly decreased these protein levels (Fig. 6A–C). The results indicated that Sal B significantly increased the expression of Smad7 in CFs induced by HG and inhibited the TGF-β1 signaling pathway. Sal B significantly increased the expression of Smad7, which was consistent with the immunofluorescence staining results (Fig. 6D, E).

### Sal B improved myocardial fibrosis by deubiquitinating Smad7 and stabilizing Smad7 protein expression

**Fig. 7:**
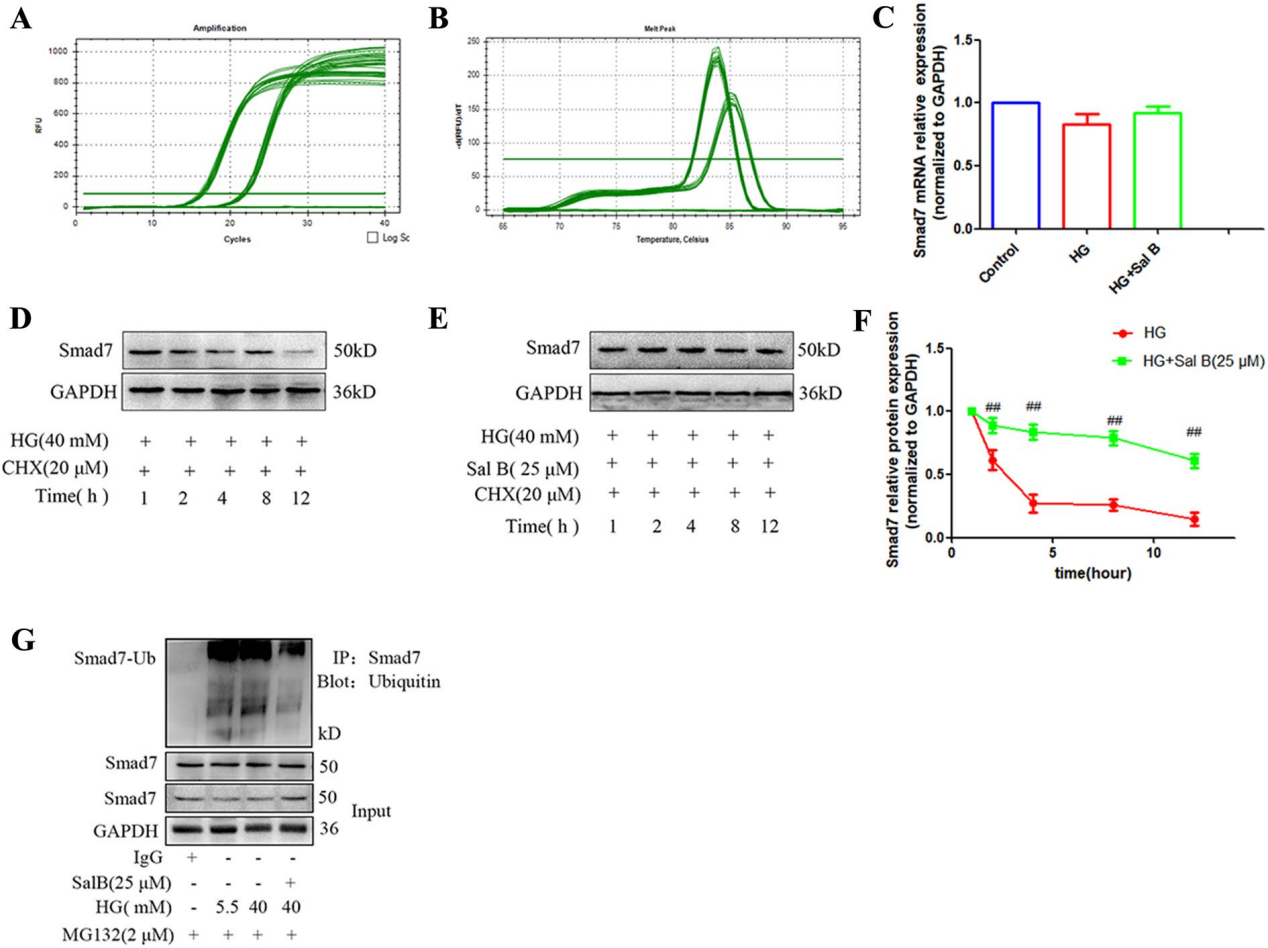
Mechanisms of Sal B on Smad7 expression. **A** Amplification curve of *Smad7* and *GAPDH*; **B** melting curve of *Smad7* and *GAPDH*; **C** quantitative analysis of Sal B-induced *Smad7* mRNA expression. The expression of *Smad7* mRNA levels was normalized to *GAPDH* (2^−△△Ct^); **D** expression level of Smad7 induced by HG at different time points; **E** Expression level of Smad7 induced by Sal B at different time points; **F** quantitative results of Smad7 protein expression. The Smad7 protein levels were normalized to GAPDH. **G** Effect of Sal B on Smad7 ubiquitination. The cell experiment was repeated three times. Data are expressed as the mean ± SD. One-way ANOVA was applied for comparisons among multiple groups, followed by Tukey’s test. ^#^*p* < 0.05, ^##^*p* < 0.01 vs. the control group, *p < 0.05, **p < 0.01 vs. the DCM group

We hypothesized that Sal B increased the expression of Smad7 although gene transcription, and qRT-PCR was used to explore the effect of Sal B on *Smad7* mRNA expression in CFs induced by HG. Compared to the control group, the mRNA expression levels of *Smad7* were slightly decreased in the HG group, but the difference was not statistically significant. After Sal B treatment, *Smad7* mRNA levels increased slightly; however, the difference was not statistically significant (Fig. 7A–C). The results showed that Sal B did not increase the mRNA expression of *Smad7*. Therefore, we speculated that Sal B may inhibit the degradation of Smad7. To determine the effect of Sal B on the stabilization of Smad7 protein levels, the protein synthesis inhibitor CHX was used to inhibit the synthesis of new proteins. The protein expression of Smad7 was detected at 1, 2, 4, 8 and 12 h after CHX treatment (20 µM). Smad7 expression gradually decreased in the HG group. The expression of Smad7 in the Sal B group was highest at 1 h and was maintained at a high level. There were significant differences in Smad7 expression at 2 h, 4 h, 8 and 12 h, indicating that Sal B significantly stabilized Smad7 expression in CFs (Fig. 7D–F). According to previous reports, myocardial fibrosis promotes the ubiquitination of Smad7 and reduces its stability, which results in decreased expression of Smad7. We confirmed that Sal B could prevent the ubiquitination of Smad7 to improve myocardial fibrosis in DCM. Immunoprecipitation was performed to observe the inhibitory effect of Sal B on the ubiquitination of Smad7. Compared to the control group, Sal B significantly reduced the ubiquitination of Smad7 (Fig. 7G). These results confirmed that Sal B acts as an antifibrotic agent by inhibiting the ubiquitination of Smad7.

## Discussion

Myocardial fibrosis is the primary pathologic feature in DCM. However, the underlying molecular mechanism remains unclear. Currently, there are no available treatments for DCM fibrosis. The discovery and development of drugs that improve myocardial fibrosis are important for DCM. Excessive collagen deposition is the main feature of myocardial fibrosis. Inhabiting myocardial fibrosis in DCM through suppressing TGF-β1 induced CFs proliferation and excessive accumulation of Col-I and III as a potential anti-fibrogenic strategies. The results of study showed a large number of deposition of collagen fibers in the myocardium tissue of DCM, which lead to the deterioration of cardiac function. It is well known that Smad7 antagonizes TGF-β1 signaling through negative-feedback actions. Studies have identified the increased expression of Smad7 in CFs as a critical strategy for improving myocardial fibrosis in DCM. Therefore, Smad7 has emerged as a potential hotspot [40]. Sal B, which is extracted from *Salvia miltiorrhiza* Bunge, has shown a potential protective effect on myocardial fibrosis induced by DCM. The results of the animal test indicated that Sal B significantly ameliorated inflammatory cell infiltration and cardiac function and decreased collagen deposition in the myocardial tissue of DCM. The results of the cell test showed that Sal B could inhibit HG-induced CF proliferation, CF migration, phenotypic transformation, and collagen secretion. These results indicate that Sal B can ameliorate myocardial fibrosis induced by DCM was correlated to Sal B significantly increased the protein expression of Smad7. However, the mechanism through which Sal B affects Smad7 expression remains unclear.

**Fig. 8:**
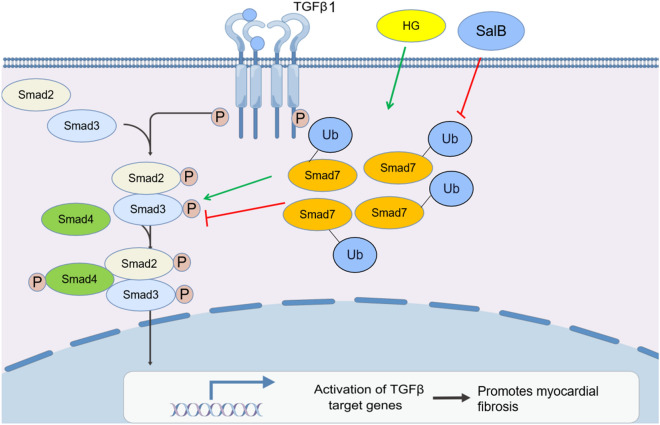
Mechanism by which Sal B improves myocardial fibrosis in DCM

After a series of experiments, we demonstrated that Sal B did not regulate Smad7 expression at the transcriptional level but instead inhibited the degradation of Smad7, thus stabilizing Smad7 protein expression. The immunoprecipitation results indicated that Sal B acts as an antifibrotic agent by inhibiting Smad7 ubiquitination (Fig. 8). However, owing to time limitations, comprehensive investigations of the impact of Sal B on the ubiquitination of Smad7 were not conducted. We speculate that Sal B may decrease the ubiquitination of Smad7 by suppressing the expression and activity of key enzymes involved in Smad7 ubiquitination, such as Smurf1 and Smurf2 [41, 42]. Alternatively, Sal B may enhance the deubiquitination of Smad7 by increasing the expression and activity of deubiquitination enzymes such as USP2 and OTUD1 [43, 44].

It is imperative to recognize a noteworthy constraint: the examination of the mechanism by which Sal B improves myocardial fibrosis in this study has exclusively focused on animal and cellular levels, specifically involving Smad7. However, the specific target directly affected by Sal B remains unclear, and further investigation will be conducted to ascertain its directly target.

## Conclusion

In summary, we demonstrated the partial amelioration of myocardial fibrosis by Sal B in a DCM mouse model by inhibiting collagen formation and deposition through the classical TGF-β/Smad pathway. Furthermore, 8 weeks of treatment with Sal B improved the structure and function of the left ventricle in diabetic mice. Therefore, Sal B is a promising therapeutic agent for DCM-induced myocardial fibrosis. However, additional clinical data are necessary to confirm the safety and effectiveness of Sal B in treating patients with DCM, and further investigations are needed to elucidate its potential mechanism.

## Data Availability

The data in this study are available from the corresponding author upon reasonable request.

## References

[1] Salvatore T, Pafundi PC, Galiero R, Albanese G, Di Martino A, Caturano A, Vetrano E, Rinaldi L, Sasso FC (2021). The diabetic cardiomyopathy: the contributing pathophysiological mechanisms. Front Med.

[2] Duan Y, Zhu W, Liu M, Ashraf M, Xu M (2017). Expression of the smad signaling pathway in the myocardium and potential therapeutic effects. Histol Histopathol.

[3] Yi-HeChen QW, Hou C-YLJ-W, Chen X-M, Zhou Q, Chen J, Wang Y-P, Li Y-G (2017). Deficiency of activin receptor-like kinase 4 alleviates myocardial infarction-induced cardiac fibrosis and preserves cardiac function. J Mol Cell Cardiol.

[4] Prachi U, Singh AP, Gupte M, Verma VK, Galindo CL, Guo Y, Zhang Q, McNamara JW, Force T, Lal H (2019). Cardiomyocyte SMAD4-dependent TGF-β signaling is essential to maintain adult heart homeostasis. JACC Basic Transl Sci.

[5] Zhao J, Wu Q, Yang T, Nie L, Liu S, Zhou J, Chen J, Jiang Z, Xiao T, Yang J, Chu C (2022). The gaseous signaling molecule SO_2_ regulates autophagy through the PI3K/AKT pathway, inhibits cardiomyocyte apoptosis and improves myocardial fibrosis in rats with type II diabetes. Korean J Physiol Pharmacol.

[6] Salvador DB, Gamba MR, Gonzalez-Jaramillo N, Gonzalez-Jaramillo V, Raguindin PF, Minder B, Gräni C, Wilhelm M, Stettler C, Doria A, Franco OH, Muka T, Bano A (2022). Diabetes and myocardial fibrosis: a systematic review and meta-analysis. JACC Cardiovasc Imaging.

[7] Li R, Qi C, Feng Q, Ding P, Kang L, Chi J (2020). Alprostadil alleviates myocardial fibrosis in rats with diabetes mellitus through the TGF-β1/Smad signaling pathway. Minerva Endocrinol.

[8] Maimaitituerxun R, Chen W, Xiang J, Kaminga AC, Wu XY, Chen L, Yang J, Liu A, Dai W (2023). Prevalence of comorbid depression and associated factors among hospitalized patients with type 2 diabetes mellitus in Hunan, China. BMC Psychiatry.

[9] Yu Y, Gu S, Li W, Sun C, Chen F, Xiao M, Wang L, Xu D, Li Y, Ding C, Xia Z, Li Y, Ye S, Xu P, Zhao B, Qin J, Chen Y-G, Lin X, Feng X-H (2017). Smad7 enables STAT3 activation and promotes pluripotency independent of TGF-β signaling. PNAS.

[10] Lee SE, Yoo J, Kim B-S, Choi HS, Han K, Kim K-A (2023). The effect of metabolic dysfunction-associated fatty liver disease and diabetic kidney disease on the risk of hospitalization of heart failure in type 2 diabetes: a retrospective cohort study. Diabetol Metab Syndr.

[11] Hongwei Y, Ruiping C, Yingyan F, Guanjun Z, Jie H, Xingyu L, Jie T, Zhenghong L, Qin G, Junfeng H, Heng Z (2019). Effect of irbesartan on the AGE-RAGE and MMP systems in a rat type 2 diabetes myocardial fibrosis model. Exp Biol Med.

[12] Mewhort HE, Turnbull JD, Meijndert HC, Ngu JM, Fedak PW (2014). Epicardial infarct repair with basic fibroblast growth factor-enhanced CorMatrix-ECM biomaterial attenuates postischemic cardiac remodeling. J Thorac Cardiovasc Surg.

[13] Hu L, Wang Y, Wan Y, Ma L, Zhao T, Li P (2021). Tangshen formula improves diabetes-associated myocardial fibrosis by inhibiting TGF-β/Smads and Wnt/β-catenin pathways. Front Med.

[14] Yan X, Zhang J, Pan L, Xue H, Zhang L, Gao X, Zhao X, Ning Y, Chen Y-G (2011). TSC-22 promotes transforming growth factor β-mediated cardiac myofibroblast differentiation by antagonizing Smad7 activity. Mol Cell Biol.

[15] Bai Y, Wang W, Yin P, Gao J, Na L, Sun Y, Wang Z, Zhang Z, Zhao C (2020). Ruxolitinib alleviates renal interstitial fibrosis in UUO mice. Int J Biol Sci.

[16] Cui X, Wang K, Zhang J, Cao ZB (2023). Aerobic exercise ameliorates myocardial fibrosis via affecting vitamin D receptor and transforming growth factor-β1 signaling in vitamin D-deficient mice. Nutrients.

[17] Fukasawa H, Yamamoto T, Togawa A, Ohashi N, Fujigaki Y, Oda T, Uchida C, Kitagawa K, Hattori T, Suzuki S, Kitagawa M, Hishida A (2004). Downregulation of Smad7 expression by ubiquitin dependent degradation contributes to renal fibrosis in obstructive nephropathy in mice. PNAS.

[18] Hao M (2022). Jatrorrhizine reduces myocardial infarction-induced apoptosis and fibrosis through inhibiting p53 and TGF-β1/Smad2/3 pathways in mice. Acta Cir Bras.

[19] Duangrat R, Parichatikanond W, Morales NP, Pinthong D, Mangmool S (2022). Sustained AT1R stimulation induces upregulation of growth factors in human cardiac fibroblasts via Gαq/TGF-β/ERK signaling that influences myocyte hypertrophy. Eur J Pharmacol.

[20] Meléndez GC, Kavanagh K, Gharraee N, Lacy JL, Goslen KH, Block M, Whitfield J, Widiapradja A, Levick SP (2023). Replacement substance P reducescardiac fibrosis in monkeys with type 2 diabetes. Biomed Pharmacother.

[21] Li CL, Liu B, Wang ZY, Xie F, Qiao W, Cheng J, Kuang JY, Wang Y, Zhang MX, Liu DS (2020). Salvianolic acid B improves myocardial function in diabetic cardiomyopathy by suppressing IGFBP3. J Mol Cell Cardiol.

[22] Cao L, Yin G, Du J, Jia R, Gao J, Shao N, Li Q, Zhu H, Zheng Y, Nie Z, Ding W, Xu G (2023). Salvianolic acid B regulates oxidative stress, autophagy and apoptosis against cyclophosphamide-induced hepatic injury in Nile Tilapia (*Oreochromis niloticus*). Animals.

[23] Jiang B, Chen J, Xu L, Gao Z, Deng Y, Wang Y, Xu F, Shen X, Guo DA (2010). Salvianolic acid B functioned as a competitive inhibitor of matrix metalloproteinase-9 and efficiently prevented cardiac remodeling. BMC Pharmacol.

[24] Yuan Q, Sun Y, Yang F, Yan D, Shen M, Jin Z, Zhan L, Liu G, Yang L, Zhou Q, Yu Z, Zhou X, Yu Y, Xu Y, Wu Q, Luo J, Hu X, Zhang C (2023). CircRNA DICAR as a novel endogenous regulator for diabetic cardiomyopathy and diabetic pyroptosis of cardiomyocytes. Signal Transduct Target Ther.

[25] Gonzalez J, Bates BA, Setoguchi S, Gerhard T, Dave CV (2023). Cardiovascular outcomes with SGLT2 inhibitors versus DPP4 inhibitors and GLP-1 receptor agonists in patients with heart failure with reduced and preserved ejection fraction. Cardiovasc Diabetol.

[26] Fang T, Ma C, Zhang Z, Sun L, Zheng N (2023). Roxadustat, a HIF-PHD inhibitor with exploitable potential on diabetes-related complications. Front Pharmacol.

[27] Luo Y, Li Y, He L, Tu H, Lin X, Zhao F, Huang Y, Wen M, Wang L, Yang Z (2023). Xinyang tablet ameliorates sepsis-induced myocardial dysfunction by regulating Beclin-1 to mediate macrophage autophagy and M2 polarization through LncSICRNT1 targeting E3 ubiquitin ligase TRAF6. Chin Med.

[28] Mei J-C, Wu AYK, Wu P-C, Cheng N-C, Tsai W-B, Yu J (2014). Three-dimensional extracellular matrix scaffolds by microfluidic fabrication for long-term spontaneously contracted cardiomyocyte culture. Tissue Eng Part A.

[29] Luo R, Lv C, Wang T, Deng X, Sima M, Guo J, Qi J, Sun W, Shen B, Li Y, Yue D (2023). A potential Chinese medicine monomer against infuenza A virus and infuenza B virus: isoquercitrin. Chin Med.

[30] Chiu CH, Lei KF, Chan YS, Ueng SW, Chen AC (2019). Real-time detection of antibiotics cytotoxicity in rabbit periosteal cells using microfluidic devices with comparison to conventional culture assays. BMC Musculoskelet Disord.

[31] Wiegand C, Hipler UC, Elsner P, Tittelbach J (2021). Keratinocyte and fibroblast wound healing in vitro is repressed by non-optimal conditions but the reparative potential can be improved by water-filtered infrared A. Biomedicines.

[32] Frangogiannis NG (2021). Cardiac fibros. Cardiovasc Res.

[33] Sakurai T, Kamiyoshi A, Takei N, Watanabe S, Sato M, Shindo T (2019). Bindel-PCR: a novel and convenient method for identifying CRISPR/Cas9-induced biallelic mutants through modified PCR using *Thermus aquaticus*. DNA Polym Sci Rep.

[34] Lei Q, Yi T, Li H, Yan Z, Lv Z, Li G, Wang Y (2020). Ubiquitin C-terminal hydrolase L1 (UCHL1) regulates postmyocardial infarction cardiac fibrosis through glucose-regulated protein of 78 kDa (GRP78). Sci Rep.

[35] Yan K, Ponnusamy M, Xin Y, Wang Q, Li P, Wang K (2018). The role of K63-linked polyubiquitination in cardiac hypertrophy. J Cell Mol Med.

[36] Asghari AA, Mahmoudabady M, Mousavi Emadi Z, Hosseini SJ, Salmani H (2022). Cardiac hypertrophy and fibrosis were attenuated by olive leaf extract treatment in a rat model of diabetes. J Food Biochem.

[37] Zhu H, Ji H, Chen W, Han L, Yu L (2022). Integrin subunit β-like 1 mediates angiotensin II-induced myocardial fibrosis by regulating the forkhead box Q1/Snail axis. Arch Biochem Biophys.

[38] Zou X, Ouyang H, Lin F, Zhang H, Yang Y, Pang D, Han R, Tang X (2022). MYBPC3 deficiency in cardiac fibroblasts drives their activation and contributes to fibrosis. Cell Death Dis.

[39] Chen PY, Shih NL, Hao WR, Chen CC, Liu JC, Sung LC (2018). Inhibitory effects of momordicine I on high-glucose-Induced cell proliferation and collagen synthesis in rat cardiac fibroblasts. Oxid Med Cell Longev.

[40] Liu J, Lu J, Zhang L, Liu Y, Zhang Y, Gao Y, Yuan X, Xiang M, Tang Q (2023). The combination of exercise and metformin inhibits the TGF-β1/Smad pathway to attenuate myocardial fibrosis in db/db mice by reducing the NF-κB-mediated inflammatory response. Biomed Pharmacother.

[41] Deng H, Yao X, Cui N, Huang S, Ge Y, Liu R, Yang X (2023). The protective effect of zinc, selenium, and chromium on myocardial fibrosis in the offspring of rats with gestational diabetes mellitus. Food Funct.

[42] Malonis RJ, Fu W, Jelcic MJ, Thompson M, Canter BS, Tsikitis M, Esteva FJ, Sánchez I (2017). RNF11 sequestration of the E3 ligase SMURF2 on membranes antagonizes SMAD7 downregulation of transforming growth factor β signaling. J Biol Chem.

[43] Kit Leng Lui S, Iyengar PV, Jaynes P, Isa ZF, Pang B, Tan TZ, Eichhorn PJ (2017). USP26 regulates TGF-β signaling by deubiquitinating and stabilizing SMAD7. EMBO Rep.

[44] Zhang Y, Qian H, Wu B, You S, Wu S, Lu S, Wang P, Cao L, Zhang N, Sun Y (2020). E3 ubiquitin ligase NEDD4 familyregulatory network in cardiovascular disease. Int J Biol Sci.

